# Crigler-Najjar syndrome: looking to the future does not make us forget the present

**DOI:** 10.1186/s13023-024-03108-x

**Published:** 2024-03-07

**Authors:** Fabiola Di Dato, Giuseppe D’Uonno, Raffaele Iorio

**Affiliations:** Department of Translational Medical Science, Section of Pediatrics, University of Naples Federico II, Via Sergio Pansini 5, 80131 Naples, Italy

**Keywords:** Gene therapy, Liver transplantation, Phenobarbital, Phototherapy

## Abstract

Recently, the safety and efficacy of gene therapy were evaluated in patients with Crigler-Najjar syndrome (CNS). Although it is a promising curative option for CNS, many doubts still persist about its long-term efficacy and safety. Furthermore, there is a risk of overlooking several unresolved problems still present in current clinical practice. This letter is a call for action on crucial open issues that remain nowadays an unmet need in the management of CNS patients.

To the Editor.

Crigler-Najjar syndrome (CNS), described for the first time in 1952 [1], is a rare autosomic recessive inherited metabolic disorder of liver caused by mutation of UGT1A1 gene that encodes the hepatic UDP-glucuronosyltransferase.

Two forms of CNS have been described: type I (CNS-I) and type II (CNS-II) [2]. CNS-I patients completely lack UGT1A1 enzyme activity and, if not promptly treated with phototherapy, develop severe neurological damage. Instead, CNS-II patients have a residual UGT1A1 enzyme activity (less than 10%) with a lower, but still present, neurological risk [3].

Recently, D’Antiga et al. evaluated the safety and efficacy of a single intravenous infusion of an adeno-associated virus (AAV) vector encoding UGT1A1 in 5 CNS patients and 3 of them, treated with a higher dose, had a decrease in bilirubin levels below 300 µmol per liter (17.5 mg/dl) and were able to stop phototherapy for the next 18 months of observation [4]. However, a complete bilirubin level normalization was never reached in any case. Although gene therapy is a promising curative option for CNS, many doubts still persist regarding its long-term effectiveness and safety. Indeed, the effectiveness time of a single infusion of AAV transgene vector is still undefined [5]. As well as, it has been reported that in patients with hemophilia, gene therapy can induce development of persistent, high-titer, cross-reactive AAV neutralizing antibodies which can preclude the possibility of further vector administrations [6]. Finally, multiple AAV-vectors infusions raise the potential threat of genotoxicity [7]. There is therefore the risk that the attractiveness of gene therapy can arouse excessive illusions in patients and lead to the neglect of several unresolved problems still present in the management of CNS. We believe that this key point is probably common to many rare diseases.

For example, a critical issue regards phenobarbital (PB) treatment. In CNS-II, the treatment with PB stimulates UGT1A1 gene transcription, increasing UDP-glucuronosyltransferase levels and reducing plasma unconjugated bilirubin concentrations by 30% or more [3]. This pharmacological treatment is historically used to discriminate between type I and type II CNS. Although it is commonly used in CNS patients, as also emerges from the D’Antiga study, it is not considered in neonatal hyperbilirubinemia guidelines [4, 8, 9]. This is probably due to the fact that, several decades ago, combined therapy of PB and phototherapy showed no advantage over phototherapy alone in the treatment of neonatal jaundice [8]. Unfortunately, since phototherapy is not easily available at home, newborns with indirect hyperbilirubinemia who have an intermediate picture between CNS-I and CNS-II may not benefit from this drug due to the lack of clear guidelines. Even the commonly used dose of 3–5 mg/kg/day has not been defined in the literature.

Newborns who after the first weeks of life have bilirubin values ​​just below 20 mg/dl represent a challenge not addressed in the available literature. They do not require phototherapy and may be discharged from the neonatal units, but pending the results of molecular analysis, such infants run the risk of having potentially neurotoxic bilirubin elevations at home. As mentioned above, for these patients there are no indications for the use of PB. In addition, there are objective difficulties in providing home phototherapy units for suspected CNS newborns after discharge, because setting up home phototherapy is expensive and equipment is not easily obtainable, not just in resource-limited settings.

Since the first description of CNS, phototherapy has been the primary measure to control bilirubin levels. Surprisingly, the first ‘guidelines’ providing principles for effective phototherapy in CNS patients were only published in June 2020, when Strauss et al. clarified which type of light source should be used, the distance of the light source from the skin, the exposed body surface area and the duration of light exposure to be used [10].

Last but not least, liver transplantation (LT) is the only definitive treatment able to normalize bilirubin levels in CNS. Currently, it does not exist any evidence-based recommendation regarding not only the indication, but also the timing of LT in CNS patients. It is not clear when in the course of the disease LT should be considered, nor what clinical and laboratory criteria (total serum bilirubin? total bilirubin/albumin ratio? loss of efficacy of phototherapy?) should be evaluated in order to refer CNS patients to liver transplantation centre. Although phototherapy has the undisputed merit of allowing long survival with at least an initial saving of neurological involvement, in addition to the risk of dehydration in young children, it inevitably undergoes a progressive loss of effectiveness over time caused by the decrease in the surface/volume ratio and the thickening of the skin during growth. Moreover, the impact on quality of life and interpersonal relationships should not be overlooked [10]. Although the issue has already been discussed [11], in the era of gene therapy, uncertainties about the real role of LT are increasing, so much so that there is a risk that CNS patients will worsen seriously while waiting for gene therapy and will not benefit from the transplant option.

In conclusion, 70 years following its first description, CNS is still a morbid and potentially fatal disorder. Transplant and non-transplant therapeutic strategies should be better defined by international evidence-based guidelines. This letter is a call for action on these crucial open issues that remain nowadays an unmet need in the management of CNS patients.

## Data Availability

Not applicable.
